# Incorporation of causality structures to complex network analysis of time-varying behaviour of multivariate time series

**DOI:** 10.1038/s41598-021-97741-2

**Published:** 2021-09-23

**Authors:** Leo Carlos-Sandberg, Christopher D. Clack

**Affiliations:** University College London, Computer Science, London, WC1E 6EA UK

**Keywords:** Complex networks, Nonlinear phenomena

## Abstract

This paper presents a new methodology for characterising the evolving behaviour of the time-varying causality between multivariate time series, from the perspective of change in the structure of the causality pattern. We propose that such evolutionary behaviour should be tracked by means of a complex network whose nodes are causality patterns and edges are transitions between those patterns of causality. In our new methodology each edge has a weight that includes the frequency of the given transition and two metrics relating to the gross and net structural change in causality pattern, which we call $\alpha$ and $\beta$. To characterise aspects of the behaviour within this network, five approaches are presented and motivated. To act as a demonstration of this methodology an application of sample data from the international oil market is presented. This example illustrates how our new methodology is able to extract information about evolving causality behaviour. For example, it reveals non-random time-varying behaviour that favours transitions resulting in predominantly similar causality patterns, and it discovers clustering of similar causality patterns and some transitional behaviour between these clusters. The example illustrates how our new methodology supports the inference that the evolution of causality in the system is related to the addition or removal of a few causality links, primarily keeping a similar causality pattern, and that the evolution is not related to some other measure such as the overall number of causality links.

## Introduction

Increasingly research is becoming interested in understanding the behaviour of interactions occurring within complex systems. Within finance and economics a popular complex system of interest is the international oil market, having been shown to be connected to market stability and stock returns^1–8^, and with oil prices from different regions influencing each other^1,9–16^. To investigate these complex systems static networks have been constructed, with each node representing a variable in the system (component/subsystem in the time series data) and each edge an interaction between variables (such as causality, correlation, etc.)^17–23^. For clarity, in this paper networks of this type with causality interactions will be referred to as “causality patterns”^13^. These networks allow for description of the system based on their features, such as the number of links present. It is well known that in many systems this static description of the causality pattern is misleading, with the interactions being time-varying^1,10,13^. This has lead to a branch of work focusing on the dynamic behaviour of these time-varying interactions. The most intuitive form of this analysis can be seen as the comparison of properties of causality patterns constructed from different segments of time, often with only a few segments choosen and being seperated by a large time gap^24–26^. As this field has grown, new techniques have been proposed that are suited to the discovery of evolutionary behaviour. In many cases this work builds on concepts constructed for univariate data. In particular many investigations of univariate time series data, which is a mature field, have been concerned with the representation of this evolution as a complex network^27–30^. A simplification of these complex networks can be described as nodes representing a state at a specific time (e.g. the value at time t of the time series) and edges representing temporally sequential states (i.e. link the node created by the current state to the one created by the state at the next time step). This construction means that the complex network will likely have fewer nodes than there are values in the time series (since multiple states will be the same) and hence properties of the network will allow for description of this evolution (e.g. clustering of certain states, particular orderings of transition, etc.: some common methods are given later in this paper). Recent work in the field of time-varying multivariate systems has expanded upon this approach to construct complex networks for the investigation of the evolution of these multivariate systems^18,31–35^. In this paper we propose a novel methodology based upon the network construction demonstrated by Jiang et al.^13^. Their network construction maps the causality patterns to nodes and the temporal ordering to weighted directed edges, such that the edge between two causality patterns encodes the number of times the first pattern transitioned to the second. This construction allows for the properties of the complex network to be used to describe the behaviour of the evolution of the causality pattern of the system.

These complex network approaches to discovering behaviour have been employed by a number of researchers^9,12,13,15,36–40^, demonstrating their usage in the field. However, there still exists opportunity for expansion within these techniques^13,33^. This paper presents a new methodology for the discovery of behaviour dictating the time-varying changes in the causality pattern of a system. This paper presents a new methodology for the discovery of behaviour dictating the time-varying changes in the causality pattern of a system, using techniques that themselves are not novel and are well known; the originality of the research is not in the novelty of the techniques but in the way the techniques are combined and applied to a field where they have not previously been used. Specifically our focus is on both the change between consecutive causality patterns and the order in which these patterns occur. This gives a richer description of the evolutionary behaviour. This is done through the proposal of a methodology for constructing complex networks that incorporates relevant information of the transitions in causality patterns. We also propose five properties/metrics of this network to demonstrate specific behaviours of interest. As a demonstrator for the methodology, the presented methods are applied to sample data from the international oil market to demonstrate their usage and to discover new descriptions of the behaviour of the interactions within this market.

## Granger causality

The analysis discussed above requires a measure of interaction between variables (time series) composing the system. This topic has seen large interest over the years and hence a vast array of methods, and variations to those methods, exist to determining these interactions^41–47^. Due to the variety of methods there are a number of different forms that this measure can take, however these commonly take the style of a value between 0 and 1, that may or may not be directed (non-directed measures can be seen as “correlation” and directed measures can be seen as “causality”), representing the strength of relation between two variables.

Previous studies applying complex networks to the evolution of the system’s interactions have primarily employed one of three interaction measures; correlation^48^, transfer entropy^46^, and Granger causality^45^. Due to our interest in the specifics of the interaction dynamics, a measure with directionality is desired, hence correlation is not appropriate. When comparing transfer entropy and Granger causality, it has been shown that in certain linear systems they are equivalent^49^. One downside of transfer entropy against Granger causality is that it has been shown to require more data^50^. This makes transfer entropy less attractive for use with our analysis that uses a sequence of overlapping small windows.

Further to this it should be noted that the methods presented in this paper can be seen as generally agnostic to the method used to determine the interactions, so long as these results are directed and ranged between 0 and 1 (with 0 being no interaction). Granger causality is a convenient basis to demonstrate new methodologies due to its simplicity and familiarity in many fields (for example economics and neuroscience^13,45,51,52^). Due to the popularity of Granger causality many extensions have been proposed^53–56^, however to allow this work to be as comprehensible as possible to a range of audiences we choose to employ the classic formulation, as is used by Jiang et al. in their similar work, and which is sufficient for the task required of it.

The Granger causality test^45^ can be described as follows: for two variables $v_x$ and $v_y$, where $v_x$ causes $v_y$ (written as $v_x \rightarrow v_y$) an unrestricted regression model is created:

1 $$\begin{aligned} v_{y, t} = \sum _{i=1}^{p} A_{i} v_{y, t-i} + \sum _{j=1}^{q} B_{j} v_{x, t-j} + \varepsilon _{t} \end{aligned}$$

where $\varepsilon _{t}$ denotes the residual error for time *t*, $i_{1..p}$ and $j_{1..q}$ denote the lag intervals, and *A* and *B* are free variables that are chosen via least squares regression^57^. This model is then compared via a hypothesis test to the restricted model:

2 $$\begin{aligned} v_{y, t} = \sum _{i=1}^{p} A_{i} v_{y, t-i} + \varepsilon _{t} \end{aligned}$$

This restricted model takes the position of the null hypothesis in the test. Therefore for a causal link to be detected the null hypothesis must be rejected.

We use the F test, a popular choice for Granger analysis^13,58,59^.

3 $$\begin{aligned} F-test = \frac{(RSS_{r} - RSS_{u})/q}{RSS_{u}/(S - p - q -1)} \end{aligned}$$

where $RSS_{r}$ and $RSS_{u}$ are the residual sum of squares for the restricted and unrestricted model respectively, and *S* denotes the sample size^13^. In this paper we use a significance level of $5\%$.

Following the work of Jiang et al.^13^ the network of causality links in a multivariate system can be captured in a causality pattern. This can be seen as an *n*-by-*n* matrix, with each element representing the causal link from *i* to *j*. The system’s evolution can therefore be captured by creating a series of causality patterns via a sliding window of fixed-length (the start of each window is one time step after the start of the previous).

## Complex networks

The analysis of non-linear behaviours, and structures, of time varying causality interactions within multivariate systems can be challenging due to the complex nature of this information. An approach to make the data more amenable to analysis is to encode the evolutionary information into a network representation^60^, as discussed in the “Introduction”.

The transfer of a time-varying multivariate system to a complex network to analyse its evolution has been employed in a number of studies^13,36,40,61,62^. Although the exact methodology for this transfer varies, the general approach can be seen as similar. In this paper we will be following the approach presented by Jiang et al.^13^ which employs Granger causality. This method can be described as constructing a complex network where the nodes represent causality patterns and the edges represent the frequency of transition between these patterns. Below we present a formal description of this general approach, to provide an unambiguous foundation for the discussion that follows. To the authors knowledge a formal specification of this nature has not been presented elsewhere.

We define:

- A set *V*, which is the set of all labelled time-series variables $v_x$ where x ranges from 1 to n inclusive. Using the notation [[*a*, *b*]] to indicate the set of all integers from *a* to *b* inclusive, we write:
  $$\begin{aligned} V = \{v_x\} \; \forall x \in [[1,n]] \end{aligned}$$
- An individual Granger causality metric $c_{x,y}$ which gives 1 if time-series variable $v_x$ Granger-causes $v_y$ and 0 otherwise. Using the previously-introduced Granger causation arrow, we write this as:
  $$\begin{aligned} c_{x,y} = {\left\{ \begin{array}{ll} 1, &{}\quad \textit{if} \; v_x \rightarrow v_y \\ 0, &{}\quad \textit{otherwise} \end{array}\right. } \end{aligned}$$
- A *time-labelled* individual Granger causality metric $c_{x,y,t}$ from time-series variable $v_x$ to $v_y$ at time *t* (where *t* is the time label of the sliding window, as previously described). Using ${\mathbb {N}}_0$ to indicate the set of natural numbers including 0, we define $c_{x,y,t}$ to be the 2-tuple $(c_{x,y}, t)$ such that the predicate $t \in {\mathbb {N}}_0$ holds. We write this as:
  $$\begin{aligned} c_{x,y,t}&= (c_{x,y}, t) \; | \; t \in {\mathbb {N}}_0 \end{aligned}$$
- A multivariate causality pattern *C*, which is the set of all individual causality metrics $c_{x,y}$ where *x* and *y* both range from 1 to *n* inclusive. We write this as:
  $$\begin{aligned} C = \{ c_{x,y} \} \; \forall {x,y} \in [[1,n]] \end{aligned}$$
- A *time-labelled* multivariate causality pattern $C_t$, which is the set of all time-labelled individual causality metrics $c_{x,y,t}$ where *x* and *y* both range from 1 to *n* inclusive and *t* is the end time of the sliding window, as previously described. Thus:
  $$\begin{aligned} C_t = \{ c_{x,y,t} \} \; \forall {x,y} \in [[1,n]] \end{aligned}$$

Each time the sliding window moves forward in time a new causality pattern is observed. If observations start at time 0 and end at time *T*, we define a set *O* to be the set of all observed time-labelled causality patterns $C_t$ where time *t* ranges from 0 to *T* inclusive. Thus:

$$\begin{aligned} O = \{C_t\} \; \forall t \in [[0,T]] \end{aligned}$$

We next define a network ${{\mathscr {N}}}$ of nodes and edges by ${{{\mathscr {N}}}} = (N, E)$ where nodes in *N* are representative causality patterns (defined below) and edges in *E* are weighted directed connections between representative causality patterns (nodes). If two or more observed causality patterns $C_{t_1}$, $C_{t_2}, \ldots C_{t_k} \; \in O$ have the same pattern of causality (albeit measured at different times) then they are represented by a single node in ${{\mathscr {N}}}$. Thus, we first define the equivalence sets of the observed time-labelled causality patterns (each equivalence set contains all observed $C_k$ that share the same underlying pattern of causality) and then define the associated labelled representative node $R_l$ to be the associated underlying causality pattern. This also requires a function to extract the underlying pattern of causality from any such $C_k$. Therefore we define.

- A function $\textit{patt}()$, which gives the set of all time-independent individual Granger causality metrics that correspond to all of the time-labelled individual Granger causality metrics in a causality pattern $C_t$. Thus:
  $$\begin{aligned} \textit{patt}(C_t) = \{c_{x,y}\} \; \forall (c_{x,y,t}) \in C_t \end{aligned}$$
- A labelled equivalence set $R^{set}_l$ with label *l* (a natural number) is a set of all 2-tuples comprising a causality pattern $C_{k}$ and label *l*, for all $C_{k}$ in the set *O* of observed causality patterns, such that all members of a given $R^{set}_l$ will have the same pattern of causality returned by the function $\textit{patt}$ (i.e a $R^{set}_l$ will exist for each unique found causality pattern and will contain all instances of that pattern observed in *O*). Thus (using “*and*” to connect multiple quantifiers, and “$\wedge$” for the logical conjunction of predicates):
  $$\begin{aligned} R^{set}_l = \{(C_k,l)\} \; \forall C_k \in O \;\;\textit{and} \;\; \forall (C_p,l), (C_q,l) \in R^{set}_l \; | \; l \in {\mathbb {N}} \; \wedge \; \textit{patt}(C_p) = \textit{patt}(C_q) \end{aligned}$$
- A labelled representative node $R_l$ (node in the network) with label *l* (a natural number) is a 2-tuple comprising (1) the underlying causality pattern of any $C_{t}$ in the equivalence set with the label *l*, and (2) the label *l*. The label provides a one-to-one mapping between each $R_l$ and its associated $R^{set}_l$ (so knowing $R_l$ implies knowledge of $R^{set}_l$):
  $$\begin{aligned} R_l = (\textit{patt}(C_t), l) \; | \; (C_t ,l)\in R^{set}_l \wedge \; l \in {\mathbb {N}} \end{aligned}$$
- The set *N* is a set of labelled representative nodes such that for each observed causality pattern $C_t$ there exists at least one representative node in *N* that contains the underlying pattern of causality of $C_t$. Thus, we write:
  $$\begin{aligned} N = \{ R_l \} \;\forall C_t \in O \;\textit{and}\; \exists R_k \in N \; | \; R_k = (\textit{patt}(C_t), k) \end{aligned}$$
- The set *E* is a set of weighted edges, each being a 3-tuple of the start node ($R_{l_1}$), the end node ($R_{l_2}$) and the weight. Initially the weight is the number of transitions from $R_{l_1}$ to $R_{l_2}$ taking place over one time step; this is commonly referred to as the frequency “*f*”. We start by defining the function $\textit{freq}()$ (where $\textit{freq}() \in {\mathbb {N}}_{0}$) to calculate the desired result—it does this by summing all transitions that exist from a causality pattern $C_t$ in $R^{set}_{l_1}$ to a causality pattern at the next time step $C_{t+1}$ in $R^{set}_{l_2}$ (i.e. the number of times one causality pattern transitions into another), Using the notation $|\{\}|$ to give the cardinality of a set, we write:
  $$\begin{aligned} \textit{freq}(R_{l_1}, R_{l_2}) = |\{((C_t,l_1) \in R^{set}_{l_1}, (C_{t+1},l_2) \in R^{set}_{l_2})\} | \;\; \forall t \in [[0,T]] \; \textit{and}\; f\in {\mathbb {N}}_0 \end{aligned}$$
  We now define the set *E* as follows (with the additional constraint that an edge does not exist in *E* if the calculated frequency is zero):
  $$\begin{aligned} E = \{(R_{l_1}, R_{l_2}, f)\} \; \forall l_1, l_2 \in [[1,|N|]] \; | \; f= \textit{freq}(R_{l_1}, R_{l_2}) \;\wedge \; f > 0 \end{aligned}$$

Following this definition the evolution of the causality between time series variables, *V*, can be expressed as the complex network ${{\mathscr {N}}}$. In this paper we are exclusively interested in expanding upon this approach to data representation in order to mine information relating to the evolution of the system, and the description of this evolution.

## New viewpoint to causality evolution

As discussed in the previous section, the general approach in construction of a complex network to represent the evolution of causality patterns only encodes information relating to frequency and temporal ordering. This approach produces a network that is easily handled by existing network approaches, and therefore generalised network analysis is often employed^60,63^. This approach to analysis can yield important results about the system and can be seen as a significant tool in the analysis of time varying causality patterns. However, these existing standard network approaches focus on frequency-weighted edges, and this does not allow for further exploration of the changes occurring between the causality patterns in the start and end nodes of each edge. This is a problem because it removes the potential to uncover complex behaviours taking place related to the change in structure of the underlying causality patterns, which may hide important information about the evolution of those causality patterns.

There do exist complex network mapping methods that take some account of the underlying structure of the system’s interactions. Of note is the work of Yu et al. who introduce a Multivariate Time Series-Dynamic Association Network (MTS-DAN) using a Directed Limited Penetrable Visibility graph (DLPVG) approach^61^. The incorporation of the underlying pattern structure in this method is achieved by using Principle Component Analysis (PCA) to produce a one-dimensional representation of each causality pattern, and then based on this representation to add new links between nodes. This approach constructs a complex network that is unweighted, directed forward in time (but not necessary temporally sequential), and contains links not associated directly with transitions. This construction therefore contains some implicit information of the causality patterns in its linkage choice. Though this network construction may be of interest in certain areas of analysis, by definition it does not contain much of the information encoded in Jiang et al.’s construction^13^, namely a guaranteed temporal ordering (if two causality patterns are sequential they will be linked) and a frequency weighting of sequential transitions (how many times one causality pattern has transitioned to a specified other).

We wish to counter these issues and present a novel methodology that is amenable to analysis and incorporates information on the change in causality pattern during evolution. Our proposed methodology takes a similar initial approach to that of Jiang et al.’s^13^ (discussed earlier), but includes a greater amount of information content through the addition of new edge weights that are specifically constructed to encode select information on the transition. We propose an extension to the complex network model presented by Jiang et al.^13^ (and discussed earlier), in regard to transitions over one time step, by adding new weights to the edges. These weights correspond to information regarding the start and end nodes, to encode information on the evolution of the causality pattern over the corresponding transition, i.e. how the causality pattern changed over one time step. The manipulation and comparison of the full causality patterns in the start and end nodes of an edge can be unwieldy where there are large numbers of times-series variables. We therefore propose to use two simple values to encode the structural change in causality pattern, as described below (where either or both aspects may occur over a single time step).

As a preliminary step, we define a metric that encodes an important informational aspect of a causality pattern. This is the arithmetic sum of all of the individual causality metrics in the causality pattern of a single node. We call this the “total causality” of a node, and it is calculated by the auxiliary function $\textit{total}()$, also used is the auxiliary function $\textit{fst}()$, which returns the first item of a 2-tuple:

$$\begin{aligned} \textit{total}(R_l) = \sum _{a,b} (c_{a,b} \in \textit{fst}(R_{l})) \end{aligned}$$

When a causality pattern changes, the overall strength of causation (the “total causality”) may or may not change. We therefore propose two new metrics to encode these two characteristics of a change in causality pattern.

1. Total difference in causality ($\alpha$): this captures the changes in individual causality metrics, regardless of the “total causality”, and we define it as the sum of the differences between corresponding individual causality metrics (each difference is squared and rooted, to make it a positive number independent of direction). This is calculated with the function $\textit{alpha}()$ as follows (where $\textit{alpha()}\in {\mathbb {Z}}$):
  $$\begin{aligned} \textit{alpha}(R_{l_1}, R_{l_2}) = \sum _{a,b} \sqrt{((c_{a,b} \in \textit{fst}(R_{l_2})) - (c_{a,b} \in \textit{fst}(R_{l_1})))^2} \end{aligned}$$
2. Net change in causality ($\beta$): this captures the overall change in “total causality”, regardless of any change in which causality links do and do not occur and we define it as the difference of the “total causality” metrics for the start and end nodes. This is calculated with the function $\textit{beta}()$ (where $\textit{beta()}\in {\mathbb {Z}}$):
  $$\begin{aligned} \textit{beta}(R_{l_1}, R_{l_2}) = \textit{total}(R_{l_2}) - \textit{total}(R_{l_1}) \end{aligned}$$

### Tri-weighted network representation

**Figure 1:**
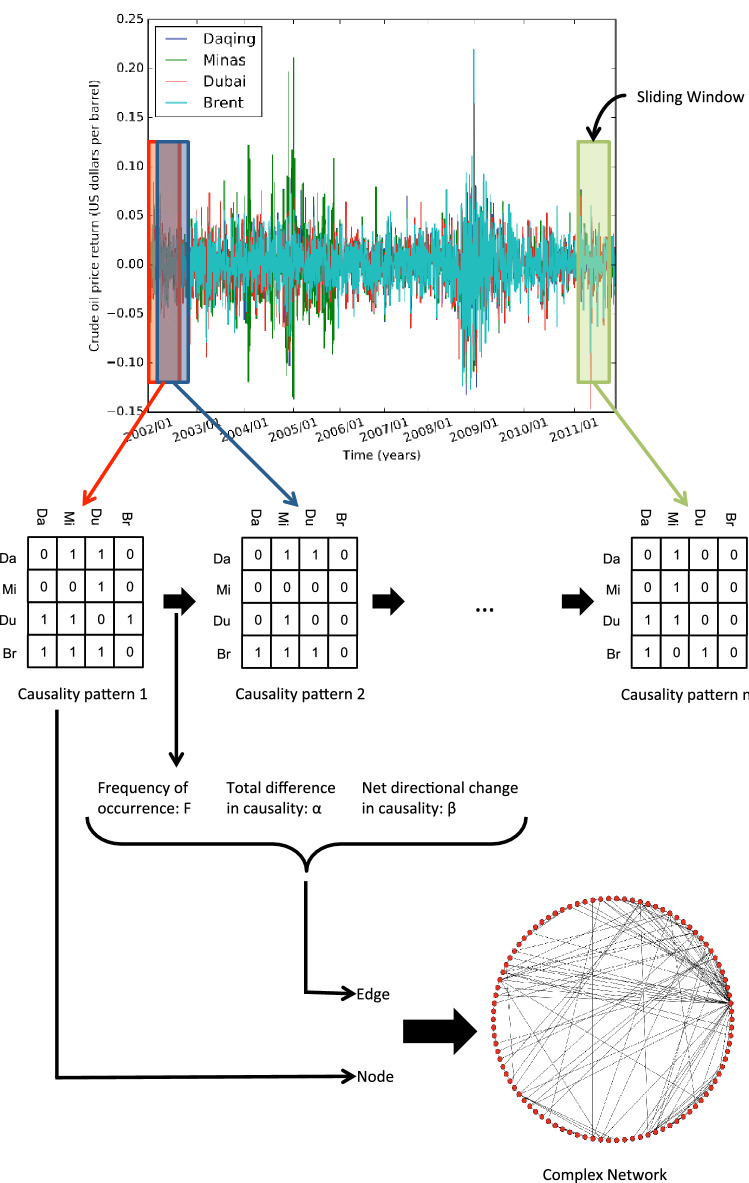
Outline of the methodology of the USIC-Network model for a sub system of the international oil market comprising the returns of the spot price variables Daqng, Minas, Dubai, and Brent. Causality patterns and complex network displayed are for representation purposes only.

Following the introduction of our two metrics we propose a new network called Underlying Structural Information Consideration Network (USIC-Network) that makes use of these metrics. This network construction takes each unique representative causality pattern as a node and takes edges between them to exist if the two causality patterns appear sequentially in the evolution. Each edge is weighted with three quantities; the frequency of transition (*f*), the Total Difference in Causality ($\alpha$), and the Net Change in Causality ($\beta$). The layout of this transformation is shown in Fig. 1.

By expanding on the earlier formal definition of a complex network representation, the USIC-Network can be defined such that the set *E* of weighted edges is modified to have a weight that is a 3-tuple containing *f*, $\alpha$ and $\beta$ (defined above). For convenience we define a labelled edge $e_{i,j}$ as follows:

$$\begin{aligned} e_{i,j}&= (R_i, R_j, (f, \alpha ,\beta )) \; |\; i,j \in {\mathbb {N}} \;\wedge \; f > 0\\&\quad \textit{where} \; f= \textit{freq}(R_{i}, R_{j}) \\&\quad \textit{and}\;\; \;\; \alpha = \textit{alpha}(R_{i}, R_{j}) \\&\quad \textit{and}\;\; \; \;\beta = \textit{beta}(R_{i}, R_{j}) \end{aligned}$$

We now rewrite the definition of *E* as:

$$\begin{aligned} E = \{e_{l_1, l_2}\} \;\; \forall l_1, l_2 \in [[1,|N|]] \end{aligned}$$

We further define three auxiliary functions *get-f()*, *get-*$\alpha$(), and *get-*$\beta$() that return the three components of an edge weight:

$$\begin{aligned}&\textit{get-f}((R_{l_{1}}, R_{l_{2}}, (f, \alpha , \beta ))) = f\\&\textit{get-}\alpha ((R_{l_{1}}, R_{l_{2}}, (f, \alpha , \beta ))) = \alpha \\&\textit{get-}\beta ((R_{l_{1}}, R_{l_{2}}, (f, \alpha , \beta ))) = \beta \\ \end{aligned}$$

With this new definition of *E* and using the previous definition of *N* we define USIC-Network $=(N, E)$ for the set of time series variables *V*.

## Analysis techniques for causal evolution

The previously mentioned studies employing complex networks for the evolution of multivariate systems by Jiang et al., Yu et al., Qi et al., Dong et al., and Yu et al.^13,36,40,61,62^ also subsequently analyse their constructed networks. This analysis takes a number of forms but primarily it determines properties and metrics of the network that can then be used to describe the evolution of the original system. This analysis can be characterised as the following types: determination of the degree of the nodes, the distribution of this degree, and the comparison of this to standard network models, such as a scale free network^13,36,40,61^; the distribution of edge weights (frequency), comparison to power law distribution, and total level of self-loops within the network (i.e. when a causality pattern does not change over a time step)^13,36^. Common network methods are used, such as the closeness of the network^40^, and the betweenness of nodes^36,40,61,62^. Clustering is also often employed for more in-depth analysis of the behaviour of the system, for example modularity-based methods^13,64^. These approaches do not explicitly allow for description of the evolution in terms of the changes in the structure of the causality pattern. Our proposed USIC-Network encodes this information allowing it to become more accessible, and hence more amenable to analysis. Numerous approaches, including many classic examples, can be applied to our USIC-Network to make use of this data, however in this section we choose to propose five properties/metrics of our USIC-Network that can be used to discover informative descriptions of the evolution of the original system, to allow for a richer overall understanding of the system. These are meant for any system that can be expressed via the USIC-Network model, however for context we motivate each approach with an example use case, demonstrating behaviour it is amenable to uncover.

### Chance of system state recurrence for stability

Interactions between financial instruments are often used when considering financial risk, and this is particularly true when portfolio construction is concerned. Within a portfolio, assets that are correlated can be seen as increasing risk, as this limits diversification and makes the portfolio less resistant to shocks: conversely assets that are negatively correlated are some times used to mitigate risk by the increase in one counteracting the decrease in the other. Similarly the causality structure within a portfolio (where known) can be carefully selected to reduce overall risk, leading to the effectiveness of the portfolio being heavily connected with this causality structure. Due to this it is important for portfolio managers that this causality structure does not change as it could force them to have to reconstruct their portfolio, and this motivates a desire for a metric for how likely a system is to maintain its current causality structure. In the context of our network this problem can be seen as a metric relating to the likelihood of a transition from a causality pattern to have no Total Difference in Causality, i.e. $\alpha = 0$. This process is often referred to as a self-loop or self transition, where the causality pattern does not change over a time step (loops that taken multiple time steps to return to their original position are not classically considered self-loops and are not of interest here).

The concept of self-loops occurs in many branches of research, for example: (1) in modularity techniques, for the purpose of separating the internal links of a community from those connecting the community to others^65–67^; (2) in Markov chains, where each state will have a probability of transitioning to itself, and is often a pre-defined value^68^; and (3) in complex networks, where the overall percentage of self-loops for a network is analysed, and specific self-loop edges with a high frequncy are discussed^36^. For the above case we are interested in a self-loop measure that can be discussed in terms of individual nodes, where each node has a probability of self-loop associated with it. Due to the variety of self-loop usage in the literature we give a definition of this metric for our USIC-Network below.

This problem can be seen as similar to that of determining the degree of a node, though unlike the degree that is often for a directed network separated out into a *in* and *out* degree we desire to separate this further into a self-loop degree. Here we present a definition of the chance of a self loop, using the following functions that are applicable to any given representative node in network ${{\mathscr {N}}}$.

- The function $k^{loop}()$ gives the number of times a representative causality pattern $R_l$ transitions over one time step to the same representative causality pattern:
  $$\begin{aligned} k^{loop}(R_l) = \textit{get-f}(e_{l,l}) \end{aligned}$$
- The function $k^{out}()$ gives the number of times a representative causality pattern $R_l$ transitions over one time step to any next representative causality pattern in *N* (including itself):
  $$\begin{aligned} k^{out}(R_l) = \sum _{R_x \in N} \textit{get-f}(e_{l, x}) \end{aligned}$$
- The probability of a self-loop occurring for a representative causality pattern $R_l$ is given by $\Omega ^{loop}_{R_l}$, defined by:
  $$\begin{aligned} \Omega ^{loop}_{R_l}= \frac{k^{loop}(R_l)}{k^{out}(R_l)} \end{aligned}$$

### Prediction of net system causality change

Causal interconnectivity within the financial markets has been shown to lead to a number of undesirable behaviours for market health when it becomes too high. This includes market behaviours such as crashes, bubbles, and other instabilities in price, leading to fallout that is both difficult and costly for regulators and governments to resolve^69^. It is therefore beneficial for market regulators to have a forecast for how the degree of causal interactions within the market are likely to change, to allow them to enact policy to limit adverse market behaviour before those changes occur. To give an indication of the change (either an increase, decrease, or no change) expected in the total causality at the next time step, it is desirable to have a one-dimensional value representing the multi-dimensional data (edges) that describes the previous changes from the relevant node.

This problem regards the out links from the node, and their associated $\beta$ weightings. For a classic network where weightings represent frequency of occurrences this problem can be seen as analogous to the out degree of the node, where the sum of the weights of all out links is calculated. Here we present a metric that gives an indication of whether causality pattern (node) is likely to increase, decrease, or maintain the same total causality, based on its history. To do this we define the following functions that are applicable to any given representative node in network ${{\mathscr {N}}}$.

- An aggregation of the previous transitions of a specific representative causality pattern $R_l$ can be found by summing the frequency of the out edges from that node, $k^{out}(R_l)$ (defined previously).
- To give knowledge of the direction of these transitions a new function $k^{weighted}()$ can be employed, that weights each edge as it is added to the sum by the sign of its $\beta$ weight (giving information relating to whether the transition increase, decreases, or does not change the total causality). This summation hence will give a value representing the average directional change in causality, which can be taken as a prediction of future directional changes in causality. First we define an auxiliary function $\textit{sign}()$ as follows:
  4 $$\begin{aligned} \textit{sign}(x) = {\left\{ \begin{array}{ll} -1, &{}\quad \textit{if} \; x < 0\\ 0, &{}\quad \textit{if}\; x = 0\\ 1, &{}\quad \textit{if}\; x > 0 \end{array}\right. } \end{aligned}$$
  And now we define $k^{weighted}()$ as follows:
  $$\begin{aligned} k^{weighted}(R_l)= \sum _{R_x \in N} (\textit{get-f}(e_{l, x}) \times \textit{sign} ( \textit{get-}\beta (e_{l, x})) \end{aligned}$$
- The value of $k^{weighted}()$ can be heavily skewed by the number of edges, and the frequency of those edges, making comparison of this value between different nodes difficult. To account for this a normalised measure of this value is proposed, $\Omega ^{directed}_{R_l}$. This is normalised by dividing $k^{weighted}()$ by the total number of out transitions given by $k^{out}()$, and hence gives a value between $-1$ and 1.
  $$\begin{aligned} \Omega ^{directed}_{R_l}= \frac{k^{weighted}(R_l)}{k^{out}(R_l)} \end{aligned}$$

### Noise perturbation from true causality pattern

In feature selection one potential aim is to determine from a set of variables those variables that Granger-cause a target variable. These variables can then be used to train a machine learning model for the purpose of forecasting the target variable. It is important for the correct subset of variables to be selected, with too large a subset increasing the cost and time of training, and too small a subset offering inferior forecasting results^70^. For feature selection it hence may be considered important to find the maximum number of potentially casual variables for a single underlying causality structure, while still minimising the total number of selected features, to reduce the number of retraining periods and present the best set of information for machine learning model to be trained on. However, many real systems, such as the financial markets, contain messy data and are susceptible to noise within their causality calculations. For example, the presence of noise can cause deviations in causality patterns and may cause a system with a singular underlying causality structure to be represented through analysis by a number of causality patterns. Therefore it can be considered beneficial to determine all potential causality patterns that may represent an underlying causal structure, allowing features to be selected from these patterns as a group, rather than just a single pattern.

Using our network representation this problem can be seen as clustering nodes based on their Total Difference in Causality ($\alpha$), with the objective of clustering together nodes with a small Total Difference in Causality. This problem can be seen as similar to those dealt with by density-based clustering methods, as this problem relates to a “small distance” measure and not a “large frequency” measure as used in alternative clustering approaches^71–73^. An initial starting point for density-based clustering is the single-linkage model, a hierarchical method that operates by grouping nodes within a given “distance” of each other and then increasing this distance till all nodes are clustered^71,74^. A notable expansion to this model was introduced by Wishart to eliminate a “chaining” effect that could lead to the linkage of widely spaced nodes via a chain of more closely connected nodes. This expansion introduced the idea of a minimum number of nodes within a set “distance” from each node in the network, a node that does not meet this minimum is then removed from a cluster^75^. The single-linkage model and the Wishart expansion act as first step for the desired clustering, however in our use case not all nodes need be clustered and due to links not existing between all nodes the colorblue usage of minimum degree does not apply. These differences in scope therefore warrant further expansion of this model for this problem.

To define this clustering property, based on some notion of measurement-based noise or variation in the network, within a USIC-Network the following steps are taken.

- We introduce a parameter $Par_{max \; \alpha }^{noise}$, which defines a maximum amount of deviation that can be expected between separate measures of the same causality pattern in a system (e.g. for a physical system a user may know that their measurement tools have an associated error and hence this metric embodies how that error translates to the measurement of a causality pattern). In the USIC-Network this measure takes the form of an $\alpha$ value, being the maximum expected $\alpha$ value between two causality patterns that could be considered the same within deviation. The exact choice of this parameter is complex and heavily system dependent, so we leave this as a user defined value (we consider more formal definitions of its exact value to be out of the scope of this paper).
- To incorporate the notion of noise that $Par_{max \; \alpha }^{noise}$ provides into the complex network, an initial USIC-Network, ${{{\mathscr {N}}}} = (N, E)$, is updated by removing both edges with an $\alpha$ greater than $Par_{max \; \alpha }^{noise}$ and any nodes that are now unconnected. This defines a new network, ${{{\mathscr {N}}}^{'}} = (N^{'}, E^{'})$, as follows:
  $$\begin{aligned} E^{'}&= \{ e_{l_1 ,l_2}\} \forall e_{l_1 ,l_2} \in E \;|\; \textit{get-}\alpha (e_{l_1 , l_2}) \le Par_{max \; \alpha }^{noise}\\ N^{'}&= \{ R_{l_1} \} \forall R_{l_1} \in N \;\textit{and} \; \exists R_{l_2} \in N \;|\; e_{l_1 , l_2} \in E^{'} \vee e_{l_2 , l_1} \in E^{'} \end{aligned}$$
- The network ${{{\mathscr {N}}}^{'}}$ now only contains transitions that are within this defined noise/deviation range. For this network we hypothesise that causality patterns (nodes) that relate to each other and are just products of noise/error will exhibit some clustering behaviour. To discover this clustering we employ a popular method known as modularity, selecting the Clauset–Newman–Moore greedy modularity maximization algorithm^76–78^. For this the edge weightings and self-loops are not considered, and a set of non-overlapping clusters are produced. Although this algorithm may not be appropriate for cases where noise clusters overlap, we assume that for most real world systems noise clusters will be adequately spaced, partly due to the binary values (quantisation) of causality, and hence overlapping clusters will not be a consideration in practice. It should also be noted that if $Par_{max \; \alpha }^{noise}$ is set such that all links are included, e.g. no consideration of $\alpha$ is taken, this approach reduces to the clustering approach employed by Jiang et al.^13^. We label each cluster produced by this approach as $\Lambda ^{noise}_{i}$, where these sets, and the set $\Lambda ^{noise}$ comprising all these clusters, can be expressed as:
  $$\begin{aligned} \Lambda ^{noise}&= \{(X, i)\} \forall X \subseteq N^{'}\\ \Lambda ^{noise}_{i}&= X \;|\; (X, i) \in \Lambda ^{noise} \end{aligned}$$

### Regimes of total causality level

The concept of regimes within the evolution of a system is a popular topic in many fields^69,79,80^. The type of grouping defining a regime can take a number of forms, in the context of our USIC-Network a potential construction for a regime is a grouping of system states (causality patterns) that have the same total causality (i.e. the level of interaction within the system is the same throughout the regime). A real world situation where this type of regime may be of interest is the behaviour between market makers during a flash crash. A particular example of this is the 2010 flash crash that has been connected to hot potato trading between market makers^81^: during a period of hot potato trading the amount of interaction between the market makers is likely to increase^82^. Therefore the regime of the total causality between market makers is likely to be different during periods of calm compared with periods of market instability such as hot potato trading, and detecting these changes in regime may be an indicator of coming instability^69,79,80^.

We are particularly interested in causal regimes whose internal edges have no net change in total causality. To this end we define a regime as a set of nodes in our USIC-Network whose internal edges all have $\beta = 0$. This means that the total causality for each node in a regime is constant, but the causality structure may not be.

For this clustering, an approach inspired by single linkage can be used, where the minimum “distance” is set as $\beta = 0$ and does not increase iteratively^71,74^. To tackle the potential problem of chaining as previously described, we introduce a parameter $Par^{regime}_{min freq}$ to represent the lowest allowed frequency level for a link. This allows for the removal of “pathways of low travel” between high travelled clustered regions. Though this can be set to some calculated value, such as the average frequency of edges, hence removing all edges below that, we choose to leave this as user selected as we feel that an appropriate value will be system-dependent. The process to find these causal regimes can be described as follows.

- From an initial USIC-Network ${{{\mathscr {N}}}} = (N, E)$, a new network can be derived where all edges with a $\beta$ value not equal to zero and a frequency value less than $Par^{regime}_{min freq}$ are removed. We call this network ${{{\mathscr {N}}}^{''}} = (N^{''}, E^{''})$ and define it as follows:
  $$\begin{aligned} E^{''}&= \{ e_{l_1 ,l_2}\} \forall e_{l_1 ,l_2} \in E \;|\; \textit{get-f}(e_{l_1 , l_2}) \ge Par^{regime}_{min freq} \;\wedge \; \textit{get-}\beta (e_{l_1 , l_2}) = 0 \\ N^{''}&= \{ R_{l_1} \} \forall R_{l_1} \in N \;\textit{and} \; \exists R_{l_2} \in N \;|\; e_{l_1 , l_2} \in E^{''} \vee e_{l_2 , l_1} \in E^{''} \end{aligned}$$
- The network ${{{\mathscr {N}}}^{''}}$ is constructed in such a way that if the desired causal regimes exist they will be components (groups of connected nodes that are not connected to the rest of the network). Therefore to find these causal regimes one extracts these components, labelling each component as a separate set $\Lambda ^{regime}_{i}$. These sets, and the set of all these regimes can be expressed as:
  $$\begin{aligned} \Lambda ^{regime}&= \{(X, i)\} \forall X \subseteq N^{''}\\ \Lambda ^{regime}_{i}&= X \;|\; (X, i) \in \Lambda ^{regime} \end{aligned}$$

### Pathways of net causality change

A broad type of structural feature that naturally arises when discussing complex networks is a “pathway”, a series of sequential nodes connected by edges, that defines some route through the network. Specific instances of pathways can be defined in numerous ways, for example in the context of our USIC-Network these definitions could be based on the *f*, $\alpha$, or $\beta$ weightings. Pathways based on *f* or $\alpha$ naturally lead to the implementation of either minimum *f* constrains (i.e. highly travelled pathways) or a maximum threshold value for $\alpha$ (i.e. closely “spaced” pathways) However, pathways based on $\beta$ can take more interesting formulations and to our knowledge have not been previously explored.

In the context of this paper $\beta$ is an interesting base for pathway construction for the previously discussed regimes (defined with edges of $\beta = 0$), with a system moving from one regime to another having to change its total causality and hence have a $\beta \ne 0$ between a node in one regime and a node in the other regime. The transition from one regime to another may occur over one time step or over many time steps. In the latter case this results in a multi-step pathway existing between the two regimes. These pathways defining a change in total causality are a general structural feature of complex causality networks, whose start and end nodes need not be members of a regime.

Here we define a type of pathway based on the $\beta$ weighting of the network. This pathway is a structure that moves the causality pattern from one level of total causality to another, with no self-loops that might be deceptive during analysis. For this initial discussion of these types of pathways we select to maintain the sign of the $\beta$ weighting throughout the pathway (i.e. a pathway will either be composed only of edges weighted as $\beta > 0$ or only of edges weighted as $\beta < 0$). This decision is well motivated for pathways between regimes, as defined above, since as soon as a $\beta =0$ edge is found we may have reached a new regime.

In defining these pathways we consider the two following constraints.

- As explained above, our initial interest is in pathways with a $\beta$ constraint: constructed of either only edges containing $\beta > 0$ or only edges containing $\beta <0$.
- For our USIC-Network (and most complex network representations) an edge is “representative” of one or many transitions at different times between observed causality patterns. As a result, these pathways are statistical in nature and don’t necessarily represent a timed sequence of transitions: it is not necessarily true that two edges in a pathway occur in the same temporal order as their representative transitions were observed. We therefore typically consider only “common” pathways by utilising a further constraint, which is to consider only edges with a frequency *f* that exceeds some threshold. The threshold will be system-dependent and experiment-dependent and we therefore express it as the user-defined parameter $Par^{path}_{min \textit{freq}}$.

Based on these constrains we present a formal definition of the pathways described above for our USIC-Network ${{{\mathscr {N}}}}= (N,E)$. We elect to split our formal definition of these pathways into two components, the pathways themselves them selves, and the first and last edges in each pathway. We label the set of all first edges (the head edges) as *H* and the set of all last edges (the tail edges) as *T* (where these are both subsets of *E*).

Employing these head and tail sets we define each pathway as a 2-tuple of a set *X* (the pathway) and a label *i* such that *X* is a subset of *E*, and there exists exactly one first edge $e_{a,b}$ in *H* and exactly one last edge $e_{c,d}$ in *T* (where neither edges are self-loops, so $b\ne a$ and $d\ne c$) such that for all edges $e_{m,n}$ in *X* three conditions must hold.

1. the edge $e_{m,n}$ must either be the first edge $e_{a,b}$ or there must exist a unique edge $e_{p,q}$ in *X* such that $e_{p,q}$ directly precedes $e_{m,n}$ (i.e. $m=q$),
2. the edge $e_{m,n}$ must either be the last edge $e_{c,d}$ or there must exist a unique edge $e_{p,q}$ in *X* such that $e_{p,q}$ directly succeeds $e_{m,n}$ (i.e. $n=p$), and
3. all edges $e_{p,q}$ must have the same $\beta$ sign, $\textit{sign}(\textit{get-}\beta (e_{p,q}))$ (where this sign will be defined via the head and tail edges).

Using the notation $\exists !$ to denote uniqueness quantification (i.e. $\exists !x$ means “there exists exactly one x”), we define the set of all such labelled pathways $\Gamma ^{H,T}$ in a given set of edges *E* with initial (head) edge in *H* and final (tail) edge in *T* as follows (with ${{{\mathscr {N}}}} = (N,E)$ assumed from here onwards):

$$\begin{aligned} \Gamma ^{H,T}&= \{(X, i)\} \forall X \subseteq E\;|\; H \subseteq X \; \wedge \; T \subseteq X \;\wedge \; i \in {\mathbb {N}}\\&\quad \wedge \; (\exists ! e_{a,b\ne a} \in H \;\textit{and} \; \exists ! e_{c,d\ne c} \in T \;\;|\; \forall e_{m,n} \in X&\;\;\;\;((m=a) \vee (\exists !e_{p,q} \in X | m=q))\\&\quad \wedge ((n=d)\vee (\exists !e_{p,q}\in X | n=p))\\&\quad \wedge (\textit{sign}(\textit{get-}\beta (e_{m,n}))=\textit{sign}(\textit{get-}\beta (e_{a,b})))\\&\quad \wedge (\textit{get-}f(e_{m,n}) \ge Par^{path}_{min \textit{freq}}) ) \end{aligned}$$

We can select a single pathway in $\Gamma ^{H,T}$ by referencing its label as follows:

$$\begin{aligned} \Gamma ^{H,T}_{i} = X\; | \;(X,i) \in \Gamma ^{H,T} \end{aligned}$$

Since we are interested in both $\beta >0$ pathways and $\beta <0$ pathways, we define four sets of edges: head edges and tail edges for $\beta >0$ pathways and head edges and tail edges for $\beta <0$ pathways. We are particularly interested in maximal pathways in a system: for example, a head node of a maximal $\beta >0$ pathway will have no in-edges with $\beta >0$ and a tail node of a maximal $\beta >0$ pathway will have no out-edges with $\beta >0$, and these constraints can be incorporated into the definitions of the sets of candidate head edges and tail edges for $\beta >0$ pathways. Similarly, we are interested in maximal $\beta <0$ pathways whose head nodes have no in-edges with $\beta <0$ and whose tail nodes have no out-edges with $\beta <0$. For all these sets the frequency constraint discussed earlier also still applies.

We label the sets of head and tail edges for pathways of $\beta >0$ as $H+$ and $T+$ respectively, and for pathways of $\beta <0$ as $H-$ and $T-$ respectively. For example, $H+$ is defined to be the set of all edges $e_{i,j}$ such that $e_{i,j}$ is in *E*, the edge has $\beta >0$, and any edge $e_{k,i}$ whose end node is the same as the start node of $e_{i,j}$ has a $\beta \not >0$. We therefore write:

$$\begin{aligned} H+&= \{ e_{i,j} \} \; | \; e_{i,j} \in E \; \wedge \; \textit{get-}f(e_{i,j}) \ge Par^{path}_{min \textit{freq}} \; \wedge \; \textit{get-}\beta (e_{i,j})>0 \; \wedge \; \forall e_{k,i} \in E \; \textit{get-}\beta (e_{k,i}) \not> 0\\ T+&= \{ e_{i,j} \} \; | \; e_{i,j} \in E \; \wedge \; \textit{get-}f(e_{i,j}) \ge Par^{path}_{min \textit{freq}} \; \wedge \; \textit{get-}\beta (e_{i,j})>0 \; \wedge \; \forall e_{k,i} \in E \; \textit{get-}\beta (e_{k,i}) \not > 0\\ H-&= \{ e_{i,j} \} \; | \; e_{i,j} \in E \; \wedge \; \textit{get-}f(e_{i,j}) \ge Par^{path}_{min \textit{freq}} \; \wedge \; \textit{get-}\beta (e_{i,j})<0 \; \wedge \; \forall e_{k,i} \in E \; \textit{get-}\beta (e_{k,i}) \not< 0\\ H-&= \{ e_{i,j} \} \; | \; e_{i,j} \in E \; \wedge \; \textit{get-}f(e_{i,j}) \ge Par^{path}_{min \textit{freq}} \; \wedge \; \textit{get-}\beta (e_{i,j})<0 \; \wedge \; \forall e_{k,i} \in E \; \textit{get-}\beta (e_{k,i}) \not < 0\\ \end{aligned}$$

Therefore two sets of pathways can be written: $\Gamma ^{H+, T+}$ is the set of maximal $\beta >0$ pathways, and $\Gamma ^{H-, T-}$ is the set of maximal $\beta <0$ pathways. Individual pathways would for example be written as $\Gamma ^{H+, T+}_{i}$ and $\Gamma ^{H-, T-}_{i}$.

For the specific problem of the algorithmic detection of pathways a simple but effective approach is to start with a node in *H* and look along connected out edges to find a new node, and then repeat this process on the new node until no new nodes can be found (where each out edge must meet the requirements specified above). In the case where a node connects directly to multiple other nodes a pathway would be constructed for each possible choice (it should be noted that this may give rise to pathways with overlapping regions). To discover the set of all pathways this process would be applied to all possible start nodes.

## Demonstration

We wish to demonstrate our proposed methods and their application via the the exploration of a real world system, as well as expanding upon the current understanding of that system. Previous research into evolving causality patterns has selected the international oil markets as a convient sample data set for demonstrating analysis techniques, due to its known interactions^13,14,83,84^. For our test data we select four authoritative spot prices Brent, Daqing, Minas, and Dubai, this data set has been previously shown to exhibit interaction dynamics by Jia et al.^14^.

**Figure 2:**
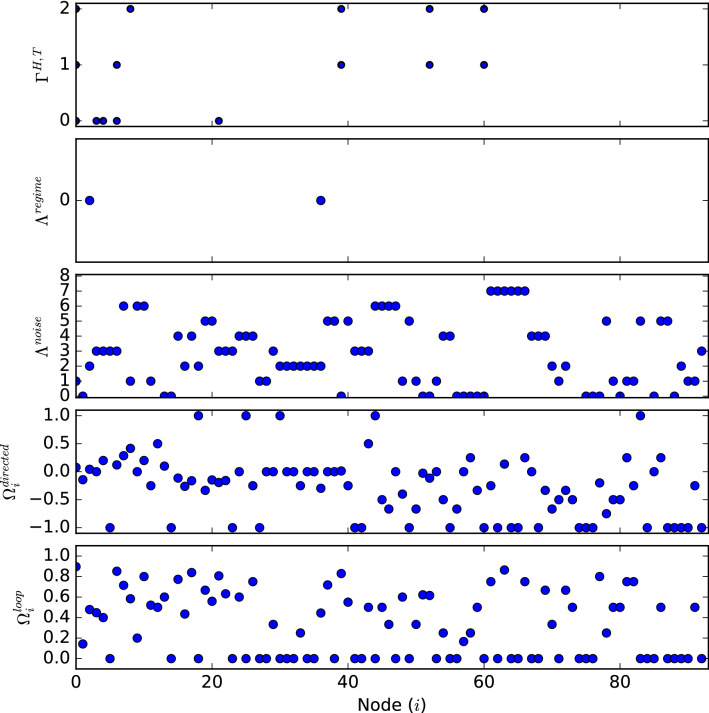
Results of analysis methods for each node on a network of Oil spot prices. $\Omega _{i}^{loop}$ and $\Omega _{i}^{directed}$ display the associated value for each node. $\Lambda ^{noise}$ and $\Lambda ^{regime}$ display the cluster labels of the node if applicable. $\Gamma ^{H, T}$ displays pathway labels of the node if applicable, these are for both $\Gamma ^{H+, T+}$ and $\Gamma ^{H-, T-}$ pathways. Note that the cluster and pathway labels starts at 0.

For our analysis we will use the daily price returns for the four series of spot prices, calculated as $r_{t} = \text {ln}(P_{t}) - \text {ln}(P_{t-1})$, with $P_{t}$ denoting the daily closing price This data covers a period from December 27th, 2001 to October 31st, 2011^13,14^. We use the Bayesian Information Criterion (BIC) to automatically selected the lag terms for our causality analysis^85^, and the Augmented Dickey-Fuller (ADF) test for stationarity, with results shown in Table 1. We transform this data set into an evolving series of causality patterns via Granger analysis, selecting a window size of 30 as discussed earlier. Using this series we then construct our USIC-Network. We then apply our analysis to this network, using parameters $Par^{noise}_{max \alpha } = 2$, $Par^{regime}_{min freq} = 1$, $Par^{path}_{min freq} = 2$, and $Par^{path}_{min len} = 4$ (this configuration was selected through experimentation), with results for each node in the network shown in Fig. 2. From our results in Fig. 2 looking at our individual analysis methods we can draw the following conclusions.

- On the whole nodes tend to favour having a chance of self-looping ($mean(\Omega ^{loop}_{i}) = 0.32$), however a few nodes have a very high chance of self-looping. This implies that there exists a few highly stable causality patterns within the evolution.
- The causal direction of nodes during transition is on average close to zero but favours negative transitions ($mean(\Omega ^{directed}_{i}) = -0.27$), showing that on the whole transitions tend to decrease the total causality of the network.
- The majority of the network belongs to one of eight noise clusters ($\Lambda ^{noise}$) with sizes of: 17, 14, 13, 12, 10, 10, 7, and 6. These clusters are defined such that no causality pattern within them may differ more then $12.5\%$. This demonstrates that the causality pattern of the international oil market transitions throughout time between groupings of very similar patterns. Implying that the specific causality patterns are non-random, and can be seen as deviations within these clusters.
- The network shows virtually no regime clustering ($\Lambda ^{regime}$), implying that causality patterns do not maintain their overall causality during transitions in non-self-loop cases. This suggests that the evolution of the system is effected by individual causal links and not the overall causality of the system.
- There only exists a few pathways of Net Change in Causality $\Gamma ^{H, T}$ that are longer than a few nodes within the network, implying that changes in net causality primarily take place over a short number of nodes. This indicates a lack of significance in Net Change in Causality in the systems evolution.

**Table 1:** Results of stationary tests using a Augmented Dickey-Fuller (ADF) test.

	ADF-statistic (3 sf)	p-value
Daqing	$-11.7$	0.001
Minas	$-20.3$	0.001
Dubai	$-51.8$	0.001
Brent	$-9.79$	0.001

A $\hbox {p-value}<0.01$ indicates the rejection of the null hypothesis for the test at a $1\%$ level.

**Figure 3:**
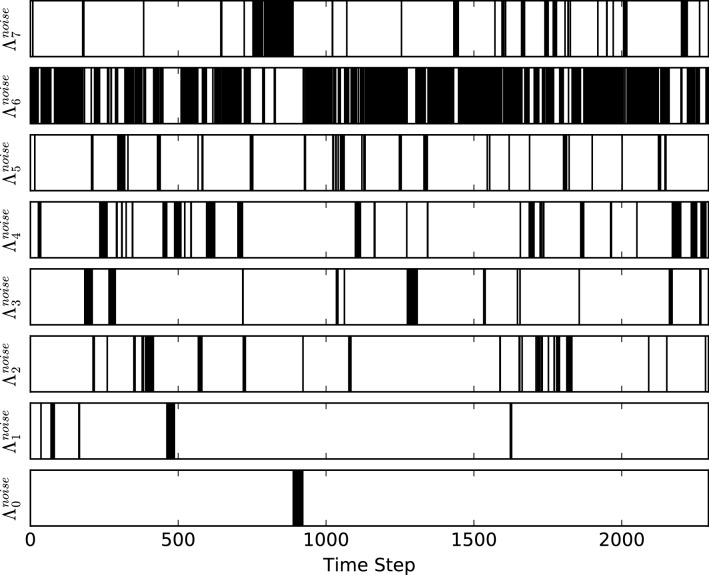
Noise cluster occupancy for the current node against time, for the whole system evolution: a black line indicates the current node is in the indicated noise cluster at the indicated time step.

To further investigate the behaviour of noise clusters within the network (from the context of the methodology), we plot the cluster the system is in at each time step during its evolution, results displayed in Fig. 3. From these results one can see that cluster 6 is very dominant within the evolution, with the system spending the majority of its time within this cluster. It can also be seen that on the whole when the system leaves this cluster it tends to stay in whichever other cluster it transitions to for a extended number of time steps. This demonstrates that the systems evolution is heavily dominated by these clusters, with causality patterns staying similar for extended time steps.

**Figure 4:**
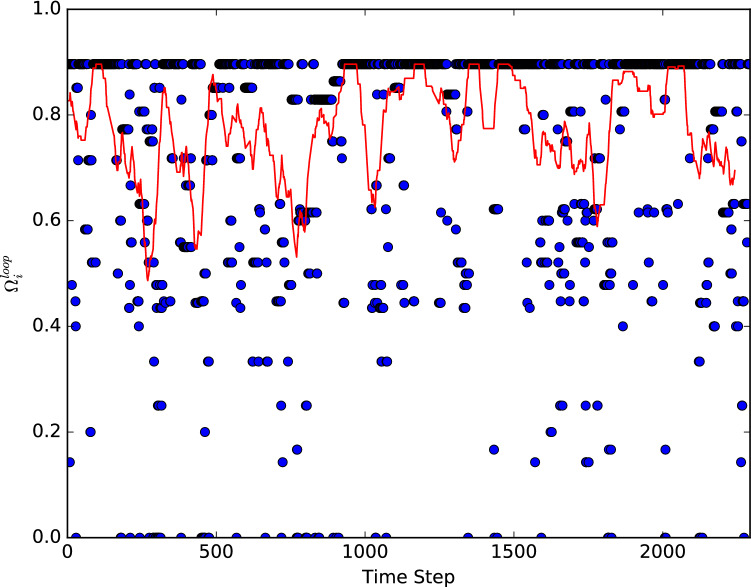
Chance of self-loop at each time step for the current node (blue circles). The red line is the rolling average with a window of 50 time steps for the self-loop chance in the evolution.

We also investigate the change in the self-loop metric during the evolution, smoothing our results with a rolling average of 50 time steps, shown in Fig. 4. The rolling window shows an approximate cyclic pattern to the evolution of this metric, where the system goes through periods of increasing self-loop chance before going through periods of decreasing self-loop chance. This implies that the system state may be moving between regions of stability, with unstable regions in-between.

These results taken together demonstrate that this evolution is highly dependent on the individual links and structure of the causality patterns and not on the overall causality. Furthermore the system favours transitions to causality patterns with a similar structure, illustrated by the evolution being able to be decomposed into a number of noise clusters. Nodes nodes with a high chance of self-looping may be considered more stable aspects of these noise clusters, with nodes with a low chance of self-looping being taken as noise/transition nodes around and between these clusters.

## Conclusion

The work presented here aims to expand the field of research, presenting a new methodology for information extraction from evolving causality networks. Exploring the evolution characteristics of time-varying causality relationships holds the potential for a deeper understanding of the dynamics of many complex multivariate systems. In this paper we encode the evolution of the interaction dynamics within a multivariate system into a series of causality patterns. We then expand on the work of authors, such as Jiang et al., by transferring these patterns to a multi-weighted directed network, the USIC-Network, capable of containing three key metrics of the evolution: frequency of transition, Total Difference in Causality, and Net Change in Causality. The addition of the latter two metrics allows for information regarding the change in the underlying causality structure to be encoded into the network. This in turn supports further analysis methods to be performed on this network. We present five novel approaches for the analysis of the evolution of interactions within a multivariate system: these methods are based on our presented network model and take advantage of the information of the underlying causality pattern. Prior to our USIC-Network model presented in this paper, this causality information was not available in a manner suitable for network analysis techniques.

To demonstrate these proposed approaches we apply them to sample data from the international oil market, due to its popularity in research and its known underlying interaction dynamics. For this data we were able to construct a behavioural description not readily discoverable with current approaches to analysis employed in the research of complex network representations of time-varying interaction dynamics. The primary aspects of this description can be divided into four linked findings: (1) the transitions over a single time step primarily result in a small change in the overall causality of the system. (2) The change in the causality pattern from a transition over a single time step changes the amount of causality in the system and not the structure of the causality (i.e. the causality pattern remains mostly similar). (3) The evolution contains clustering, specifically eight clusters wherein the causality pattern of every member node is very similar (differing by no more than two causality links). (4) The evolution goes through cycles of “high” and “low” stability (likelihood of self-loop), implying the existence of and movement between favoured causality patterns. These results as a whole can be taken to infer that the system favours a few causality (patterns plus some deviation around these) that it moves between through the addition or subtraction of a couple causality links. Therefore the structure/layout of the causality of the market is important, with the overall amount of causality in the system playing a less important role for the evolution.

The main purpose of this research is to expand upon the existing methodologies on multivariate systems, breaking away from limitations of studies depending on a static understanding of causality. This is a complex field, and in this paper we offer new techniques and analysis methods to assist those undertaking this research.

## Data Availability

Data accessed from https://www.sciencedirect.com/science/article/pii/S0140988315001000^14^.
